# Bioassay Directed Isolation and Biological Evaluation of Compounds Isolated from *Rubus fairholmianus* Gard.

**DOI:** 10.1155/2014/204340

**Published:** 2014-09-01

**Authors:** Blassan Plackal George, Parimelazhagan Thangaraj, Cheruthazhakkatt Sulaiman, Shanmughavel Piramanayagam, Sathish Kumar Ramaswamy

**Affiliations:** ^1^Bioprospecting Laboratory, Department of Botany, School of Life Sciences, Bharathiar University, Coimbatore, Tamil Nadu 641 046, India; ^2^Phytochemistry Division, Centre for Medicinal Plants Research, Arya Vaidya Sala, Kottakkal 676 503, India; ^3^Department of Bioinformatics, Bharathiar University, Coimbatore, Tamil Nadu 641 046, India

## Abstract

The *in vitro* and *in silico* analysis of *Rubus fairholmianus* acetone extract for antioxidant, antiproliferative, and anti-inflammatory activity led to the isolation of six compounds. Amongst all the six isolated compounds tested, 1-(2-hydroxyphenyl)-4-methylpentan-1-one (compound **1**) and 2-[(3-methylbutoxy) carbonyl] benzoic acid (compound **2**) were found to be more active in inhibiting BRCA and COX target proteins, which also showed the better results for DPPH and ABTS radical scavenging assays. The promising results of this investigation emphasize the importance of using *R. fairholmianus* in the treatment of radical generated disorders mainly cancer and other inflammatory diseases.

## 1. Introduction

The genus* Rubus* is very diverse, includes over 750 species in 12 subgenera, and is found on all continents except Antarctica [1]. Due to useful ethnomedicinal and pharmacological properties,* Rubus* species has been used in folk medicine [2]. The leaf extract of* R. fairholmianus* (*R. moluccanus* L.) collected in the early morning has been used by* Koch-Rajbongshi* and* Rangia* tribes to reduce headache by [3]. The leaves have been reported to possess insecticidal properties; fruits are edible and stimulant [4]. The total phenolic content and antioxidant and analgesic potential of* R. fairholmianus* extracts have been evaluated by George et al. [5].* R. fairholmianus* root acetone extract showed significant anti-inflammatory and wound healing properties which may be due to the presence of analogues of quercetin and other related polyphenolic compounds [6].

The literature showed that the berries (*Rubus* sp.) of Rosaceae family have been reported for their strong antioxidant and pharmacological properties [5–9] and various bioactive free radical scavenging compounds were isolated [10–14]. Berry fruits are characterized by a high content and wide diversity of bioactive compounds such as phenolic compounds, organic acids, tannins, anthocyanins, and flavonoids [15]. Oxidative stress is caused by an imbalance between antioxidant systems and the production of oxidants, which is associated with many diseases like cancers, cardiovascular diseases, and inflammation related disorders [16]. A large number of naturally occurring antioxidant compounds have been identified from different plant sources, as free radical or active oxygen scavengers [17, 18]. Antioxidants protect organisms against free radicals and they are vital to neutralize the destruction caused by the radicals with a sufficient supply of antioxidants [19].

Molecular docking is an approach to help researchers to screen a large set of small molecules by orienting and scoring them in the binding site of target proteins. Top ranked compounds have been tested* in vitro* and further they may become lead compounds for drug development. The Glide score, number of H-bonds, distance of bonds, interacted protein residues, and ligand atom were observed from docking studies. This docking study helps us in understanding the binding mode of the isolated compounds with the target proteins.

In the present scenario the antioxidant researchers are mainly focusing on the identification and isolation of new natural antioxidant compounds from different plants species, since it can protect the human body from various free radical generated disorders. A survey of literature revealed that the phytochemical aspects of this plant have not been evaluated. This study aimed to investigate the antioxidant activities of different extracts of* R. fairholmianus*. This study focuses on the isolation and identification of bioactive antioxidant compounds from* R. fairholmianus* through chromatographic techniques based on the activity guided fractionation of the root acetone extract. The isolated compounds then checked for its antioxidant potential. Further, we adopted docking studies to identify inhibiting activity of the compounds against BRACA and COX proteins.

## 2. Materials and Methods

### 2.1. Plant Collection and Extraction

The fresh plant parts of* R. fairholmianus* were collected from Marayoor Shola forest, Kerala, India, during the month of September 2010. The collected plant material was identified and authenticated by (Voucher specimen number BSI/SRC/5/23/2010-11/Tech. 1657) Botanical Survey of India, Southern Circle, Coimbatore, Tamil Nadu. The roots were extracted successively using acetone in a soxhlet apparatus for 72 hours. The extract was concentrated to dryness under reduced pressure in a rotary evaporator.

### 2.2. Determination of* In Vitro* Antioxidant Activities

The different fractions and isolated compounds from root acetone extracts of* R. fairholmianus* were analyzed for their antioxidant activity using DPPH assay and ABTS assay.

#### 2.2.1. DPPH Radical Scavenging Activity

The DPPH assay was done as per the method described by Blois [20]. Negative control was prepared by adding 100 *μ*L of methanol in 5 mL of 0.1 mM methanolic solution of DPPH^∙^. The tubes were allowed to stand for 20 minutes at 27°C. Radical scavenging activity of the samples was expressed as IC_50_ which is the concentration of the sample required to inhibit 50% of DPPH^∙^ concentration.

#### 2.2.2. Trolox Equivalent Antioxidant Capacity (TEAC) Assay

The total antioxidant activity was measured by ABTS radical cation decolourization assay according to Re et al. [21]. Triplicate determinations were made at each dilution of the standard, and the percentage inhibition was calculated against the blank (ethanol) with an absorbance at 734 nm and then was plotted as a function of Trolox concentration. The unit of total antioxidant activity (TAA) is defined as the concentration of Trolox having equivalent antioxidant activity expressed as *μ*M/g sample extracts.

### 2.3. Chromatographic Separation of the Root Acetone Extract

Based on the* in vitro* antioxidant studies, RFRA (*R. fairholmianus* root acetone) showed maximum antioxidant activity and it was selected for the further isolation of phytoconstituents. The preliminary screening was done using thin layer chromatography by toluene: ethyl acetate: acetic acid (6 : 3 : 0.5) as mobile phase. The extract (50 g) was adsorbed on activated silica (230–400 mesh). The column (90 × 5 cm) was packed with activated silica gel (230–400 mesh) in toluene and it was eluted with 400 mL of each of different solvents such toluene (100%), toluene : ethyl acetate (9 : 1, 7 : 3, 6 : 4, 5 : 5, 4 : 6, 2 : 8), ethyl acetate : chloroform (9 : 1, 7 : 3, 5 : 5, 3 : 7, 1 : 9), chloroform : methanol (8 : 2, 6 : 4, 5 : 5, 4 : 6), diethyl ether (100%), ethanol (100%), methanol (100%), acetic acid (2%), and acetone (100%). A total of 183 fractions were collected and the fractions with similar chemical profiles were pooled based on TLC analysis.

Based on the TLC, the fractions with similar banding pattern were clubbed and 16 fractions were obtained. The various fractions such as F1 (34–37), F2 (38–54), F3 (55–58), F4 (59–72), F5 (76–80), F6 (84–88), F7 (89–110), F8 (111–116), F9 (121–126), F10 (132–136), F11 (137–141), F12 (142), F13 (143–147), F14 (148–151), F15 (159–162), and F16 (163–183) were subjected to antioxidant studies. The most active fractions such as F5, F7, F9, and F16 were selected for further isolation process.

The 4 active fractions further yielded 6 subfractions: methanol and ethyl acetate subfractions of F5 (F5M and F5EA), chloroform subfractions of F7 and F9 (F7C and F9C), and methanol and diethyl ether subfractions of 16 (F16M and F16DE). All these fractions were analysed by TLC, HPLC, and so forth.

### 2.4. Purification of Isolated Compounds

The isolated compounds were purified by semipreparative TLC and preparative HPLC. The bands visualized under UV at 254 nm and 366 nm were scrapped out and dissolved in respective solvents. The time based collection was performed using preparative fraction collector on Agilent 1200 high pressure liquid chromatographic system equipped with prep pump, a rheodyne injector, and diode array detector in combination with Chem32, Chemstation software. C18-scalar (5 *μ*m, 4.6 × 150 mm) Agilent column was used for separation with a binary gradient elution. Mobile phase: 0.1% acetic acid in acetonitrile (A) : methanol : water (60 : 40) (B) 0–2: 70% A, 2–5 60% A, 5–10: 50% A, 10–20 70% A. The flow rate was maintained as 15 mL/minute.

### 2.5. Characterization of Isolated Compounds

#### 2.5.1. UV Spectroscopy

The UV-visible spectrum of the isolated compound in HPLC grade methanol was recorded using a Shimadzu 160 A UV-visible spectrophotometer at a range of 280–400 nm.

#### 2.5.2. Fourier Transform Infrared Spectrometry (FTIR)

The FTIR spectrum was recorded using a Nicolet 5700 (Thermo electron, Madison, WI, USA) spectrometer at room temperature. The bioactive compound was dissolved in dimethyl sulfoxide (DMSO) and scanned in the range of 4000–400 cm^−1^.

#### 2.5.3. Nuclear Magnetic Resonance Spectroscopy (NMR)

NMR spectra were recorded on a Bruker DRX 500 NMR instrument operating at 500 MHz for ^1^H and 125 MHz for ^13^C at room temperature. A region from 0 to 12 ppm for ^1^H and 0 to 200 ppm for ^13^C was employed. Signals were referred to as the internal standard tetramethylsilane (TMS). About 10 mg of the sample dissolved in 0.5 mL of CDCl_3_ was used for recording the spectra.

#### 2.5.4. Mass Spectrometry

LC-ESI-MS analysis was conducted on Agilent 6520 accurate mass Q-TOF LC/MS coupled with Agilent LC 1200 equipped with Extend-C18 column of 1.8 *μ*m, 2.1 × 50 mm. Gradient elution was performed with methanol (solvent A, 70%) and 0.1% formic acid (solvent B, 30%) at a constant flow rate of 0.3 mL/min. Column temperature was maintained at 30°C. The MS analysis was performed using ESI in the negative mode. The conditions for mass spectrometry were as follows: drying gas (nitrogen) flow 5 L/min; nebulizer pressure 50 psig; drying gas temperature 325°C; capillary voltage + 3000 V; fragmentor volt 250 V; Oct Rf Vpp 750 V.

### 2.6. Molecular Docking of Bioactive Compounds against BRCA and COX Proteins

Molecular docking has become an increasingly important tool for drug discovery. It is a useful vehicle for investigating the interaction of a protein receptor with its ligand and revealing their binding mechanism as demonstrated by a series of studies [22–27]. We have employed flexible docking strategies in this study to identify the most suitable ligand binging sites in the BRCA and COX target proteins. Flexible molecular docking operations require no prior knowledge of the ligand conformation and such an approach becomes necessary if there is no useful information on the conformation of a particular ligand.

#### 2.6.1. Protein Retrieval and Preparation

Three-dimensional structures of BRCA1, BRCA2, COX-1, and COX-2 proteins were obtained from PDB database (PDB id: 1T15, 3EU7, 1EQH, 3LN1) (PDB http://www.rcsb.org/). The retrieved protein has been prepared for docking using Protein Preparation Wizard [28]. Finally the grid was generated for the prepared proteins.

#### 2.6.2. Inhibitory Molecules Retrieval and Preparation

Inhibitory molecules were retrieved from PubChem database in 3D SDF format. The compounds such as cyclophosphamide, letrazole, doxorubicin, paclitaxel, and tamoxifen (PubChem id: CID 2907, CID 3902, CID 31703, CID 36314, CID 2733526) are the commercially available standard drugs for the treatment of cancer; diclofenac, indomethacin, paracetamol, aspirin, and morphine (PubChem id: CID 3033, CID 3715, CID 1983, CID 2244, CID 5288826) are the NSAIDs used commonly for the treatment of inflammation related diseases. About 6 compounds such as 3(imino methyl)-2,4-dimethyl phenol, isopentyl benzoate or 3-methylpentyl benzoate, 4-methylpentyl benzoate, 2-(isopentyloxycarbonyl) benzoic acid, 2-(5-methylhexyl) benzoic acid, and 1-(2-hydroxyphenyl)-4-methyl pentan-1-one isolated from* R. fairholmianus* root acetone extract were screened for their inhibitory activity against BRCA and COX proteins. Retrieved molecules were prepared for docking using LigPrep module [29]. Finally the prepared compounds were used for docking with BRCA and COX proteins.

#### 2.6.3. Molecular Docking of Target Proteins with Inhibitory Molecules

The docking was performed using Glide module [30–32], through blind docking approach in which commercially available drugs and newly isolated compounds were docked against BRCA and COX proteins. Docked complex was examined with an emphasis on visual rather than numerical appraisal, so we used XP visualizer.

### 2.7. Statistical Analysis

All the values are expressed as mean ± SEM. The values are analyzed using one-way ANOVA and the significance of the difference between means was determined by Duncan's, Tukey Kramer multiple comparisons using SPSS.

## 3. Results and Discussion

### 3.1. *In Vitro* Antioxidant Activity

**Figure 1:**
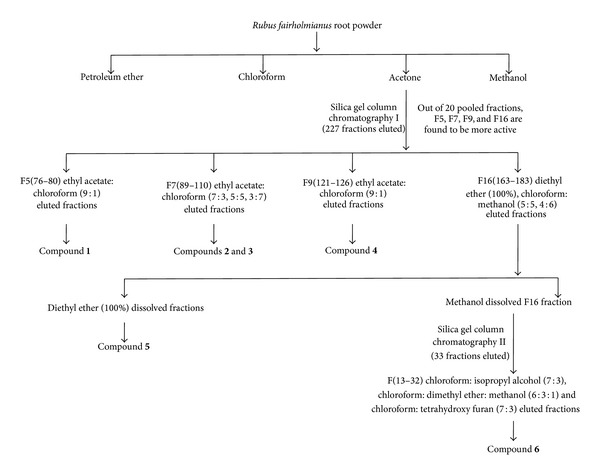
Schematic representation showing the isolation procedure of bioactive compounds from* R. fairholmianus*.

Figure 1 schematically represents the isolation procedure of bioactive compounds; all the compounds reported in this study were isolated for the first time from* R. fairholmianus*. The antioxidant activity of different fractions and isolated compounds was evaluated using DPPH and ABTS radical scavenging assays. The results are given in Table 1. Among the different fractions, F9 showed highest scavenging activity against DPPH (IC_50_ value 3.61). The IC_50_ values of the isolated compounds ranged between 12.35 and 3.23 *μ*g/mL. Compound** 1** (1-(2-hydroxyphenyl)-4-methylpentan-1-one) showed maximum DPPH radical scavenging activity with IC_50_ 3.23 *μ*g/mL. The ABTS radical scavenging activities of the fractions ranged between 1140.74 and 4974.72 *μ*M TE/g. The isolated compounds also showed commendable ABTS radical scavenging activities, with highest value for 2-[(3-methylbutoxy) carbonyl] benzoic acid (compound** 2**) (9233.20 *μ*M TE/g).

No previous reports are available on DPPH radical scavenging activity of this species. The phenolic compounds in berries have been well characterized as natural antioxidants, which are believed to play a major role in certain health benefits. The naturally occurring phenolic antioxidants encompass a diverse range of chemical classes that protect against the damage caused by ROS to DNA and membrane and cellular components [33]. The strong antioxidant activities of* R. fairholmianus* may be due to the phenolic compounds. Previously, Vadivelan et al. [34] reported the antioxidant properties of* R. ellipticus* root. The root methanol extracts showed strongest radical scavenging activity (IC_50_: 12.2 *μ*g/mL) against DPPH^∙^ and (IC_50_: 2.5 *μ*g/mL) against ABTS radical. Comparing these results, the DPPH and ABTS radical scavenging activities of the isolated column fractions and compounds of* R. fairholmianus* are highly significant and are almost equivalent and more than that of the standards. The DPPH radical scavenging activity of an allied species* R. sanctus* has also been reported, which was found to scavenge the DPPH radical by 83.27% compared to vitamin C and BHT (97.15 and 96.47%) [35]. Comparing the literatures, the isolated compounds and fractions of* R. fairholmianus* root acetone extract have a commendable antioxidant activity in the tested assays.

### 3.2. Identification of the Isolated Bioactive Compounds

The structure and molecular formula of the isolated compounds are shown in Table 2.

*Compound* ***1***. The fraction F5 yielded yellowish white amorphous solid (10.2 mg) after recrystallization on methanol. UV (methanol) *λ*max nm: 280; IR (KBR) cm^−1^: 3438.53, 1729.41, 1614.15, 1279.43. ^1^H NMR (CDCl_3_) *δ* 7.73, 7.73, 7.54, 7.53, 4.8, 4.3, 1.55, 2.07, 0.98, 0.99. ^13^C NMR: *δ* 169.43, 160.01, 134.16, 132.66, 130.61, 122.11, 73.56, 43.56, 31.46, 29.49, 20.91. MS (*m/z*) [M-H]: 191.98. The compound was identified as 1-(2-hydroxy phenyl)-4-methyl pentan-1-one. 

*Compound* ***2***. Fraction F7 presented brownish crystalline solid (8.46 mg) on evaporation. UV (methanol) *λ*max nm: 290. IR (KBR) cm^−1^: 3430.90, 2926.30, 1725.88, 1278.61. ^1^H NMR (CDCl_3_): *δ* 8.03, 7.92, 7.89, 7.71, 7.54, 7.51, 4.30, 4.29, 2.05, 2.04, 1.73, 1.70, 0.98. ^13^C NMR: *δ* 167.71, 125.69, 128.84, 66.55. The molecular formula of this compound was established based on the mass spectrum MS (*m/z*) [M-H]: 235.12. The structure was confirmed as cis-2-(isopentyloxycarbonyl) benzoic acid. 

*Compound* ***3***. [2-(5-Methylhexyl) benzoic acid] 6.79 mg, white amorphous solid recrystallised on evaporation of chloroform from fraction 7 (F7). UV (methanol) *λ*max nm: 290; IR (KBR) cm^−1^: 3427.20, 2926.85, 1728.35, 1626.05, 1281.20 and 1127.82. ^1^H NMR (CDCl_3_): *δ* 7.71, 7.70, 7.69, 7.53, 7.52, 7.51, 7.50. 4.24, 4.23, 4.22, 4.20, 4.19, 4.18. ^13^C NMR: *δ* 167.73, 142.39, 132.49, 132.36, 130.88, 130.86, 128.80, 66.19, 38.76, 36.68, 35.50, 32.50, 28.80. MS (*m/z*) [M-H]: 219.19. 

*Compound* ***4***. [4-Methylpentyl benzoate] 6.18 mg, white amorphous crystals from the diethyl ether fraction of F9. UV (methanol) *λ*max nm: 280; IR (KBR) cm^−1^: 2958.00, 1727.47, 1462.12, 1280.11 and 1125.88. ^1^H NMR (CDCl_3_): *δ* 7.71, 7.70, 7.69, 7.53, 7.51, 4.26, 4.23, 4.21, 1.70, 1.68, 1.67, 1.40, 0.99, 0.98. 0.94, 0.92. ^13^C NMR: *δ* 167.76, 133.96, 132.48, 128.81, 68.18, 38.76, 30.28, 14.04. MS (*m/z*) [M-H]: 205.23. 

*Compound* ***5***. [3-(Iminomethyl)-2,4-dimethylphenol] white crystals (10.58 mg) obtained from F16; UV (methanol) *λ*max nm: 272; IR (KBR) cm^−1^: 3451.40, 2926.73, 1576.91, 1436.96, 1218.30 and 1077.45. ^1^H NMR (CDCl_3_): *δ* 8.41, 6.80, 6.57, 5.47, 4.27, 4.28, 4.38, 4.38, 2.46, 2.32. ^13^C NMR: *δ* 118.48, 115.13, 45.07, 20.80, 149.49, 135.97, 134.19, 126.46, 18.24. MS (*m/z*) [M-H]: 148.08. 

*Compound* ***6***. [3-Methyl benzoate] (12.08 mg) was obtained as yellow amorphous crystals from methanolic fraction of F16. UV (methanol) *λ*max nm: 275; IR (KBR) cm^−1^: 3525.78, 2958.46, 1727.90, 1282.65 and 1127.62. ^1^H NMR (CDCl_3_): *δ* 8.03, 7.51, 7.92, 4.29, 4.32, 2.06, 2.07, 1.68, 1.73, 0.94, 0.99. ^13^C NMR: *δ* 167.71, 125.69, 128.84, 130.90, 135.99, 30.58, 27.74, 19.18, 19.15. MS (*m/z*) [M-H]: 191.10.

There are many reports available on the phytochemical characterization and activity guided studies of the related species of* Rubus.* Most of the compounds isolated from the* Rubus* species belong to cyanidin type compounds, anthocyanins, and tannins. Ruiz et al. [36] reported the presence of total anthocyanins (0.34–0.92 *μ*mol/g), monomeric anthocyanins (0.31–1.16 *μ*mol/g), and cyanidin-3-sambubioside in the fruits of* R. geoides*. Kubota et al. [37] isolated and purified the anthocyanins: cyanidin-3-O-glucoside and pelargonidin-3-O-glucoside from* R. croceacanthus* fruits and pelargonidin-3-O-glucoside and pelargonidin-3-Orutinoside from* R. sieboldii* fruits. Porter and coworkers [38] reported the minor flavonoid components in* R. idaeus*. Estupiñan et al. [39], Vasco et al. [40], Garzón et al. [41], Mertz et al. [11], Kim et al. [42], Ku and Mun [43], Bae et al. [44], Deighton et al. [45], Wyzgoski et al. [46], Dossett et al. [47], Ling et al. [48], Dossett et al. [49], Tulio et al. [50], and Tian et al. [51] reported the presence of cyanidin-3-glucoside and cyanidin-3-rutinoside, cyanidin-3-sambubioside, cyanidin-3-xylosylrutinoside, pelargonidin-3-glucoside, and pelargonidin-3-rutinoside, cyanidin glycoside, lambertianin C and sanguiin H-6, and 2 ellagitannins in various* Rubus* species. Bowen-Forbes et al. [52] reported the presence of eight hydroxyursane type compounds: euscaphic acid, 1-b-hydroxyeuscaphic acid, hyptatic acid B, 19a-hydroxyasiatic acid, trachelosperogenin, 4-epi-nigaichigoside F1, nigaichigoside F1, and trachelosperoside B-1 in the ethyl acetate extract of* R. rosifolius* fruits.

### 3.3. Molecular Docking of Isolated Compounds against BRCA and COX Proteins

**Figure 2:**
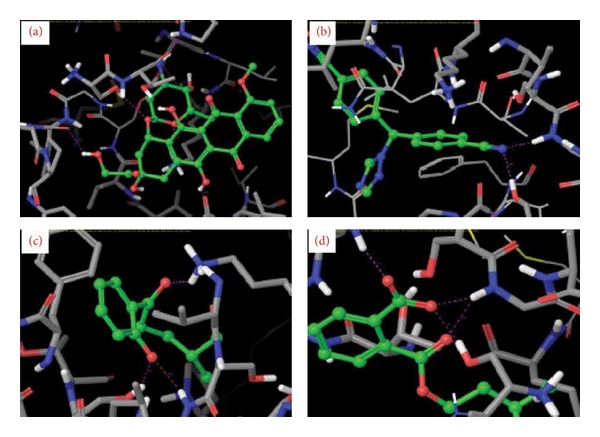
Close view of BRCA1 docked with (a) doxorubicin, (b) letrazole, (c) 1-(2-hydroxyphenyl)-4-methylpentan-1-one, and (d) 2-[(3-methylbutoxy)carbonyl]benzoic acid. Isolated compounds (green color ball stick mode) docked with target protein BRCA1 (stick mode) through hydrogen bond interaction (pink color dot).

**Figure 3:**
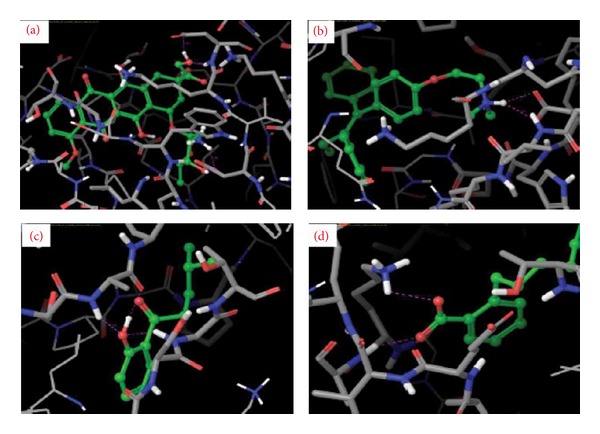
Close view of BRCA2 docked with (a) doxorubicin, (b) tamoxifen (c), 1-(2-hydroxyphenyl)-4-methylpentan-1-one, and (d) 4-methylpentyl benzoate. Isolated compounds (green color ball stick mode) docked with target protein BRCA2 (stick mode) through hydrogen bond interaction (pink color dot).

Isolated compounds and standard drugs for cancer treatment were docked against BRCA1. The docking score and hydrogen bond interactions of the compounds are showed in Table 3. 1-(2-Hydroxyphenyl)-4-methylpentan-1-one (*G* score: −4.7) has high binding with active site of BRCA1 protein followed by 2-[(3-methylbutoxy) carbonyl] benzoic acid (*G* score: −3.86). The standard drugs such as doxorubicin and letrazole also showed strong binding affinity (*G* score: −4.82 and −4.18, resp.). The docking results of BRCA1 revealed that the mode of molecular interactions of the isolated compounds and standard drugs was almost similar (Figure 2). Meanwhile, 1-(2-hydroxyphenyl)-4-methylpentan-1-one and 4-methylpentyl benzoate also showed fairly good binding affinities towards the target protein BRCA2 with a *G* score of −4.5 and −3.8, respectively. 2-[(3-Methylbutoxy) carbonyl] benzoic acid also docked with a moderate *G* score (−3.1); the binding affinities were found to be more than that of the standards letrazole and paclitaxel (*G* score: −3.0 and −2.9). The standard drug doxorubicin showed a *G* score of −11.1 followed by tamoxifen (*G* score: −5.9). Among the 6 isolated compounds, only three compounds were found to have good docking with target protein. The molecular interaction and docking score of the compounds are shown in Table 4 and Figure 3. The molecular docking results showed that the isolated compound 1-(2-hydroxyphenyl)-4-methylpentan-1-one is highly significant in binding (*G* score: −4.7) and formed three hydrogen bonds against SER 6: H (HG), GLY 1656: (H) H, and LYS 1702 (H) HZ2 residues of BRCA1. The residues involved in binding were more or less similar with that of standard drugs. The docking score of 2-[(3-methylbutoxy) carbonyl] benzoic acid, 4-methylpentyl benzoate, 2-(5-methylhexyl) benzoic acid, 3-(iminomethyl)-2,4-dimethylphenol, and 3-methylbutyl benzoate compounds ranged between −3.4 and −3.83 and the docking score of the standard drugs falls between −2.0 and −4.82. Docking score and H-bond interaction of all 6 isolated compounds were closely related to standard drugs. The docking results of BRCA2 with the isolated compounds 1-(2-hydroxyphenyl)-4-methylpentan-1-one and 4-methylpentyl benzoate are significant. 1-(2-Hydroxyphenyl)-4-methylpentan-1-one formed three hydrogen bonds with the ALA 874: (O) O, ALA874: (H) H, and GLY1166: (H) H residues of BRCA2 protein and 4-methylpentyl benzoate formed 2 hydrogen bonds with the ALA 1063: (H) H and LYS 1062 (H) HZ2 residues of BRCA2. Some of the binding residues were common in both standard drugs and the isolated compounds. The *G* score of isolated compounds ranged between −4.5 and −3.1, whereas the standard drugs were in between −11.1 and −3.2. BRCA1 and BRCA2 predispose individuals to breast, ovarian, and other cancers. These proteins are required for the maintenance of genetic stability and have function in DNA damage [53]. Previously, Raja et al. [54] studied the docking analysis of mangrove-derived compounds and revealed that, among six compounds triterpenoid, stigma sterol, and pyrethrin were efficient in destroying BRCA1. Saravanakumar and coworkers [55] proposed the docking of fungal metabolites against BRCA1. The docking score of phthalic acid was lowest (−13.71 kcal/mol) followed by 2,3-dihydro-1H-inden-2-yl acetate and 3-methylcyclopentan-1-ol having the value of −11.11 and −9.08, respectively. However, there is no report on the docking of isolated compounds from* R. fairholmianus* against BRCA proteins. So the information gained from this study will be useful for further development of novel breast cancer inhibitors.

The binding pocket of BRCA1 contains two chains with two fragments (BCRT1 and BCRT2). BRCA1 is interacting with protein c-terminal helicase 1. The highly conserved C-terminal BRCT repeats that function as a phosphoserine/phosphothreonine-binding module. Clapperton et al. [56] reported the X-ray crystal structure at a resolution of 1.85 Å of the BRCA1 tandem BRCT domains in complex with a phosphorylated peptide representing the minimal interacting region of the DEAH-box helicase BACH1. The structure revealed the determinants of this novel class of BRCA1 binding events. Transcription/antitumor protein BRCA2 contains two chains with a fragment c-terminal WD40 domain having residues 835–1186. The synonym is PALB2, Fanconi anemia group n protein. 19-meric peptides have been identified from BRCA2 protein. The second fragment interacts with PALB2 having residues 21–39. The early onset protein (BRCA2) is central to the repair of DNA damage. BRCA2 recruits recombinase RAD51 to sites of damage, regulates its assembly into nucleoprotein filaments, and thereby promotes homologous recombination. Localization of BRCA2 to nuclear foci requires its association with the partner and localizer of BRCA2 (PALB2), mutations which are associated with cancer predisposition, as well as subtype n of Fanconi anaemia. Oliver et al. [57] have determined the structure of the PALB2 carboxy-terminal beta-propeller domain in complex with a BRCA2 peptide.

**Figure 4:**
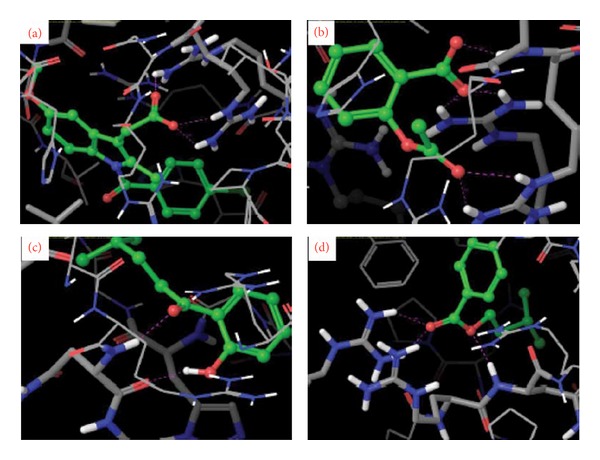
Close view of COX-1 docked with (a) indomethacin, (b) aspirin, (c) 1-(2-hydroxyphenyl)-4-methylpentan-1-one, and (d) 2-[(3-methylbutoxy)carbonyl]benzoic acid. Isolated compounds (green color ball stick mode) docked with target protein COX-1 (stick mode) through hydrogen bond interaction (pink color dot).

**Figure 5:**
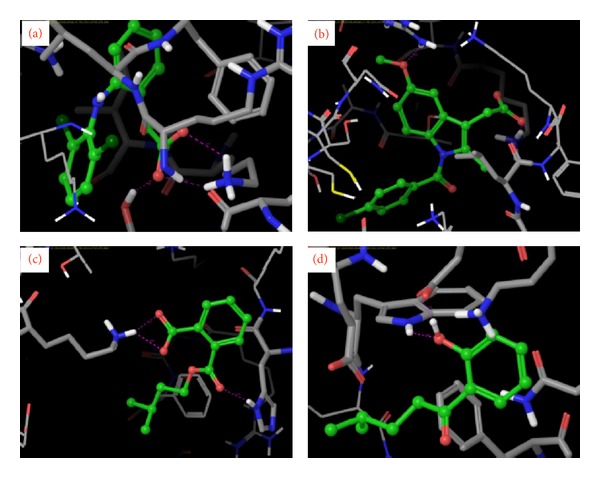
Close view of COX-2 docked with (a) diclofenac, (b) indomethacin, (c) 2-[(3-methylbutoxy) carbonyl] benzoic acid, and (d) 1-(2-hydroxyphenyl)-4-methylpentan-1-one. Isolated compounds (green color ball stick mode) docked with target protein COX-2 (stick mode) through hydrogen bond interaction (pink color dot).

The molecular docking insights provide isolated compounds and standard drugs were docked against the COX-1 and COX-2 proteins. The results of COX-1 docking showed, among 6 compounds, only 4 compounds were found to be docked firmly with the target protein. The docking score and the interaction of the compounds seem to be highly significant. 1-(2-Hydroxyphenyl)-4-methylpentan-1-one and 2-[(3-methylbutoxy) carbonyl] benzoic acid showed strong binding affinities with *G* score of −5.8 and −4.4, respectively. The *G* score of the compounds were found to be higher than the majority of the standard drugs used. However, indomethacin also showed good results (*G* score −6.67). The *G* score of the isolated compounds 2-(5-methylhexyl) benzoic acid and 3-(iminomethyl)-2,4-dimethylphenol were −3.43 and −3.23, respectively, whereas the *G* scores of the standard drugs such as aspirin, diclofenac, paracetamol, and morphine were −5.65, −3.77, −3.41, and −3.36, respectively (Table 5). The topmost interacted compounds against target proteins are showed in Figure 4. The binding of isolated compounds and standard drugs to the active sites of COX-2 protein is shown in Figure 5. The docking score and the interaction of the compounds were tabulated (Table 6). The *G* score of the compounds 2-[(3-methylbutoxy) carbonyl] benzoic acid and 1-(2-hydroxyphenyl)-4-methylpentan-1-one was −2.9 and −2.4. The *G* score of the standard drug diclofenac is −3.4.

The results of the COX-1 docking with the isolated compounds are found to be significant. The *G* score of the three isolated compounds (−3.23 to −5.28) was higher than standard drugs such as diclofenac, paracetamol, and morphine (−3.77, −3.41, and −3.36). The hydrogen bond interaction of 2-[(3-methylbutoxy) carbonyl] benzoic acid and diclofenac seems to be similar. 1-(2-Hydroxyphenyl)-4-methylpentan-1-one formed two hydrogen bonds with the ASN 375: (H) H and ASN 375: (O) O residues of COX-1 and 2-[(3-methylbutoxy) carbonyl] benzoic acid formed three hydrogen bonds with the ARG 374: (H) HH21, ARG 374 (H) HH12, and ASN 375: (H) H residues of COX-2. The docking results of isolated compounds with COX-2 are significant when compared with the standard drugs. The *G* score of the compounds ranged between −2.9 and −0.4; 2-[(3-methylbutoxy) carbonyl] benzoic acid had the highest *G* score in the isolated compounds, bound with HID 228: (H) HD1, LYS 543: (H) HZ1, and LYS 543: (H) HZ1 residues of the COX-2 protein. The binding residues (LYS 543: (H) HZ3, LYS 543: (H) HZ2, and THR 547: (H) HG1) of the diclofenac were also similar to 2-[(3-methylbutoxy) carbonyl] benzoic acid. Recent studies have shown that stellatin has COX-1 and COX-2 inhibitory activities which was isolated from* Dysophylla stellata*. Docking study revealed the binding orientations of stellatin and its derivatives into COX-1 and COX-2 and thereby helps to design potent inhibitors [58]. Olgen et al. [59] reported the COX inhibitory activities of indomethacin derivatives (*N*-substituted indole-2-carboxylic acid esters). All the derivatives were shown to be docked at the site where intact flurbiprofen was embedded for COX-1 and s-58 (1-phenylsulphonamide-3-trifluoromethyl-5-*para*-bromophenylpyrazole) for COX-2. Three series of spiro derivatives have been synthesized and docked to COX-2 by Amin et al. [60]. The results showed nearest value of indomethacin. Abdel-Aziz et al. [61] designed a group of cyclic imides (1–13) and evaluated its selective COX-2 inhibition. The molecular docking study showed that the CH_3_O substituents of 5b were inserted firmly to COX-2, where the O-atoms of such group underwent a H-bonding interaction with HIS 90 (2.43, 2.83 Å), ARG 513 (2.89 Å), and TYR 355 (3.34 Å). This revealed a similar binding mode to SC-558, a selective COX-2 inhibitor. Basile and coworkers [62] have synthesised 14 sulfonilamidothiopyrimidone derivatives. Compounds** 2**–**5** were able to fit into the active site of COX-2 with highest scores and interaction energies. Furthermore, compound** 2**, which showed an inhibition of around 50% on PGE2 production, was best scored. El-Sayed et al. [63] designed and synthesized new arylhydrazone derivatives and a series of 1,5-diphenyl pyrazoles from 1-(4-chlorophenyl)-4,4,4-trifuorobutane-1,3-dione 1. The designed compounds docked into the COX-2. Docking study of the synthesized compounds** 2**f,** 6**a, and** 6**d into COX-2 revealed a similar binding mode to SC-558, a selective COX-2 inhibitor.

Synonym of prostaglandin H2 synthase-1 is COX-1. Nonsteroidal anti-inflammatory drugs block prostanoid biosynthesis by inhibiting prostaglandin H (2) synthase. Selinsky et al. [64] reported the crystal structure of COX-1 complex with the inhibitors ibuprofen, methyl flurbiprofen, flurbiprofen, and alclofenac at resolutions ranging from 2.6 to 2.75 A. These structures allow direct comparison of enzyme complexes with reversible competitive inhibitors (ibuprofen and methyl flurbiprofen) and slow tight-binding inhibitors (alclofenac and flurbiprofen). All the inhibitors bind to the same site and adopt similar conformations. In all four complexes, the enzyme structure is essentially unchanged, exhibiting only minimal differences in the inhibitor binding site. The different apparent modes of NSAID binding may result from differences in the speed and efficiency with which inhibitors can disturb the hydrogen bonding network around Arg-120 and Tyr-355. Chou [65] studied the various methods adopted to understand the molecular mechanism of proteins and conduct structure based drug design by timely using the updated information of newly found sequences. He has also reported three main strategies developed in structural bioinformatics, that is, pure energetic approach, heuristic approach, and homology modeling approach, as well as their underlying principles, in a review. These approaches help to understand the action mechanisms of proteins and stimulating the course of drug discovery. In the literature, the binding pocket of a protein receptor to a ligand is usually defined by those residues that have at least one heavy atom (i.e., an atom other than hydrogen) with a distance < 5 Å from a heavy atom of the ligand. Such a criterion was originally used to define the binding pocket of ATP in the Cdk5-Nck5a^∗^ complex [66] and has later proved quite useful in identifying functional domains and stimulating the relevant truncation experiments. The similar approach has also been used to define the binding pockets of many other receptor-ligand interactions important for drug design.

Computational molecular docking is a useful tool for investigating the interaction of a target protein receptor with its ligand and revealing their binding mechanism as demonstrated by various studies [22–27]. In this study well known docking program Glide module is used to dock the isolated compounds of* R. fairholmianus* root acetone and the standard drugs to the active pockets of BRCA and COX target proteins to study the mode of interaction and inhibitory properties. Many marvelous biological functions in proteins and DNA and their profound dynamic mechanisms, such as switch between active and inactive states [67], cooperative effects [68], and allosteric transition [69], can be revealed by studying their internal motions [70]. Likewise, to really understand the action mechanism between a drug compound and its target protein, we should consider not only the static binding structure but also the dynamical information obtained by simulating their internal motions or dynamic process. We will consider this in our future studies.

## 4. Conclusions

The present study describes the bioactivity guided isolation, structure elucidation and antioxidant activities, and molecular docking of 6 compounds: 1-(2-hydroxy phenyl)-4-methyl pentan-1-one (**1**), cis-2-(isopentyloxycarbonyl) benzoic acid (**2**), 2-(5-methylhexyl) benzoic acid (**3**), 4-methylpentyl benzoate (**4**), 3-(iminomethyl)-2,4-dimethylphenol (**5**), and isopentyl benzoate or 3-methyl benzoate (**6**) from the root acetone extract of* R. fairholmianus*. No previous reports are available on the compound isolation and other bioactivity studies of this plant. The investigation describes the isolated compounds from acetone extract of* R. fairholmianus* root docked to the active pockets of BRCA and COX proteins to predict analogous binding mode of the BRCA and COX inhibitors. The results revealed that the compounds were docked more firmly and inhibited the breast cancer related BRCA1 and BRCA2 oncoproteins and COX-2 and COX-1 inflammatory proteins. Among the 6 compounds, 1-(2-hydroxyphenyl)-4-methylpentan-1-one and 2-[(3-methylbutoxy) carbonyl] benzoic acid were found to be more active in inhibiting BRCA and COX proteins, which also revealed the better results for antioxidant assays. Therefore, these compounds may be considered as the lead compounds for further development of therapeutic drugs.

## Figures and Tables

**Table 1:** DPPH and ABTS radical scavenging activities of fractions and compounds of *R. fairholmianus*.

Fractions and compounds	DPPH radical scavenging activity (IC_50_: *µ*g/mL)	ABTS radical cation scavenging activity (*µ*M Trolox equivalents/g extract)
**1**	14.73	2240.99 ± 32.55
**2**	17.95	1363.49 ± 23.38
**3**	18.70	3749.60 ± 5.85
**4**	20.31	3074.61 ± 35.56
**5**	7.16	3496.48 ± 51.96
**6**	18.01	4478.60 ± 35.56
**7**	9.55	3374.98 ± 23.38
**8**	25.56	2335.49 ± 40.92
**9**	3.61	4650.72 ± 15.47
**10**	15.36	4377.35 ± 11.69
**11**	21.97	2234.24 ± 5.85
**12**	17.61	2122.86 ± 32.55
**13**	27.79	1140.74 ± 30.93
**14**	24.63	4765.47 ± 47.85
**15**	16.39	4974.72 ± 45.66
**16**	13.41	3860.98 ± 42.15
Compound **1**	3.23	5344.45 ± 24.94
Compound **2**	5.09	9233.20 ± 54.32^a^
Compound **3**	10.76	6421.23 ± 84.46^d^
Compound **4**	8.57	8754.78 ± 42.29^b^
Compound **5**	12.35	5656.32 ± 38.53
Compound **6**	9.43	7455.54 ± 62.98^c^
Butylated hydroxy toluene (BHT)	13.18	—
Butylated hydroxy anisole (BHA)	4.88	—
Rutin	5.81	—
Quercetin	4.12	—

Values are mean of triplicate determination (*n* = 3)  ±  standard deviation. Statistically significant at *P* < 0.05 where a > b > c > d.

**Table 2:** Structure of the isolated compounds.

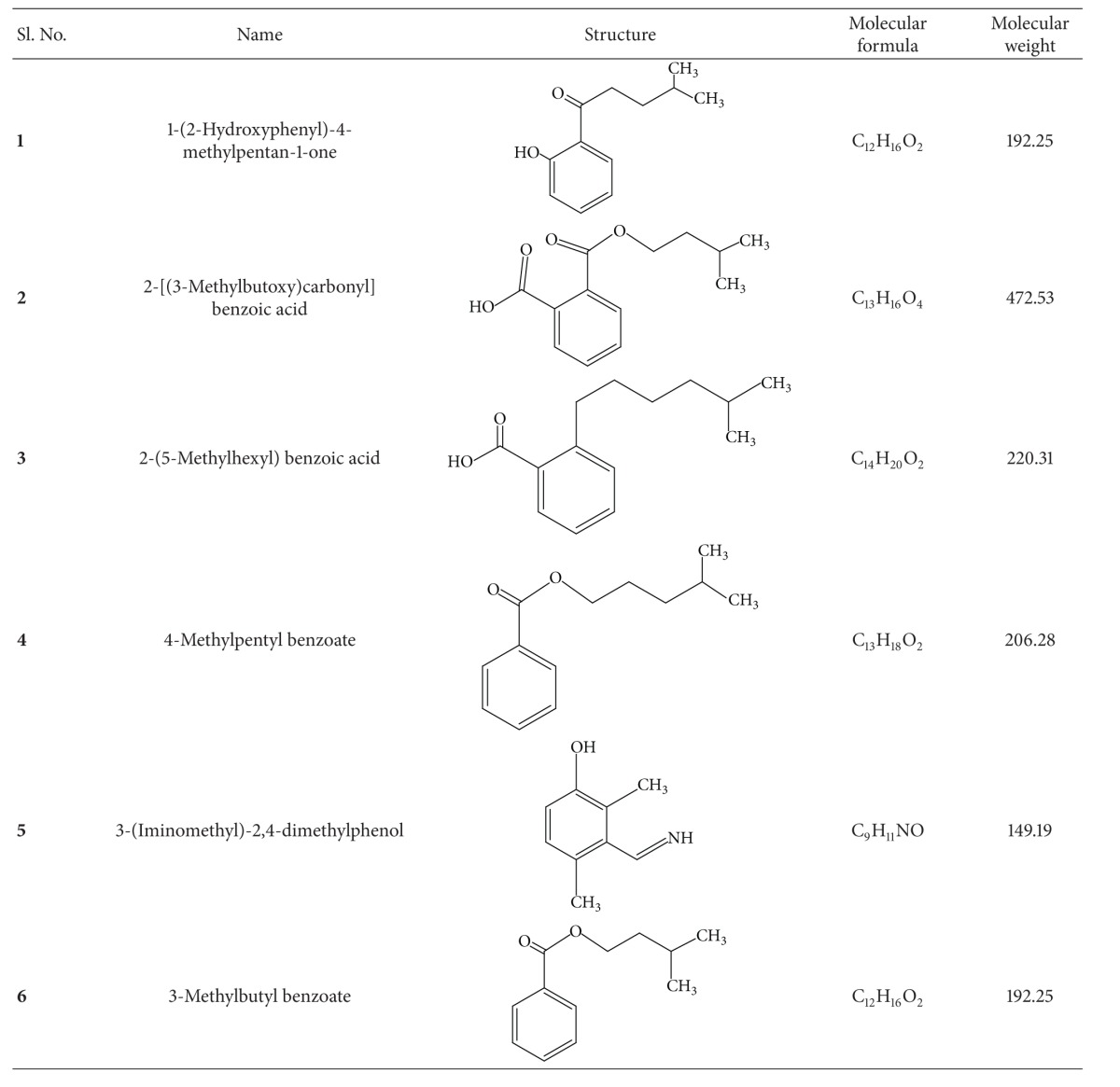

**Table 3:** Docking score and H-bond interaction of ligands with BRCA1 protein (1T15).

Ligand	*Gs*core	Number of H-bonds	Distance (Å)	Protein atom	Ligand atom
Doxorubicin	−4.82	5	2.449 2.418 2.470 2.203 2.013	SER 6: H (HG) LEU1657: (O) O ASN1678: (O) O PRO9: (O) O LYS1702: (H) HZ2	O H H H O

Letrazole	−4.18	2	2.109 2.056	GLY 1656, (H) H SER 6: H (HG)	N N

Cyclophosphamide	−3.38	3	2.172 1.944 1.753	GLY 1656, (H) H SER 6: H (HG) LYS1702: (H) HZ2	O O O

Tamoxifen	−2	1	2.004	SER 6: H (HG)	N

1-(2-Hydroxyphenyl)-4-methylpentan-1-one	−4.7	3	2.223 2.098 2.012	LYS1702: (H) HZ2 GLY 1656, (H) H SER 6: H (HG)	O O O

2-[(3-Methylbutoxy)carbonyl] benzoic acid	−3.86	5	2.257 2.142 2.070 1.916 1.709	SER 6: H (HG) GLY 1656, (H) H GLY 1656, (H) H SER 6: H (HG) LYS1702: (H) HZ2	O O O O O

4-Methylpentyl benzoate	−3.83	3	2.032 1.776 1.677	GLY 1656, (H) H SER 6: H (HG) LYS1702: (H) HZ2	O O O

2-(5-Methylhexyl) benzoic acid	−3.7	1	2.066	LEU1701: (H) H	O

3-(Iminomethyl)-2,4-dimethylphenol	−3.6	3	2.477 2.009 1.999	LYS1702: (H) HZ2 PRO9: (O) O LEU1701: (H) H	N H O

3-Methylbutyl benzoate	−3.4	1	1.947	LYS1702: (H) H	O

Glide score, number of H-bond interactions, distance of H-bond interaction, and involved protein atom and ligand atom in interaction of isolated and standard drugs with BRCA1 protein obtained from docking studies.

**Table 4:** Docking score and H-bond interaction of ligands with BRCA2 protein (3EU7).

Ligand	*G* score	Number of H-bonds	Distance (Å)	Protein atom	Ligand atom
Doxorubicin	−11.1	4	2.043 1.994 1.921 1.926 1.843	LYS1062: (H) HZ3 ASP1122: (O) OD1 ASP1125: (H) H ASP1125: (O) OD2 ASP1122: (O) OD1	O H O H H

Tamoxifen	−5.9	2	2.125 2.022	ASP1122: (O) OD2 ASP1122: (O) OD2	H H

Letrazole	−3.0	2	2.179 2.185	VAL969: (H) H HID1061: (H) HD1	N N

Cyclophosphamide	−3.2	2	2.211 1.923	LYS1062: (H) HZ3 GLU1018: (H) H	O O

Paclitaxel	−2.9	4	2.429 1.933 1.819 1.408	LYS1062: (H) HZ3 LYS1062: (H) HZ3 GLY971: (O) O HID1061: (O) O	O O H H

1-(2-Hydroxyphenyl)-4-methylpentan-1-one	−4.5	3	2.382 2.287 1.850	GLY1166: (H) H ALA874: (H) H ALA874: (O) O	O O H

4-Methylpentyl benzoate	−3.8	2	2.149 2.215	ALA1063: (H) H LYS1062: (H) HZ2	O O

2-[(3-Methylbutoxy)carbonyl] benzoic acid	−3.1	—	—	—	—

Glide score, number of H-bond interactions, distance of H-bond interaction, and involved protein atom and ligand atom in interaction of isolated and standard drugs with BRCA2 protein obtained from docking studies.

**Table 5:** Docking score and H-bond interaction of ligands with COX1 protein (1EQH).

Ligand	*G* score	Number of H-bonds	Distance (Å)	Protein atom	Ligand atom
Indomethacin	−6.67	3	2.230 2.271 1.986	ARG 374: (H) HH22 ARG 374: (H) HH21 ARG 374: (H) HE	O O O

Aspirin	−5.65	5	2.066 2.043 2.199 1.857 1.781	ARG 374: (H) HH22 ASN 375: (H) H ARG 374: (H) HE ARG 374: (H) HH12 ARG 374: (H) HH21	O O O O O

Diclofenac	−3.77	3	2.238 2.017 1.824	ARG 374: (H) HH22 ASN 375: (H) H ARG 374: (H) HH22	O O O

Paracetamol	−3.41	2	2.139 1.936	ARG 374: (H) HH21 GLY225: (O) O	O H

Morphine	−3.36	1	1.984	ARG 374: (H) HH22	O

1-(2-Hydroxyphenyl)-4-methylpentan-1-one	−5.28	2	1.964 1.734	ASN 375: (H) H ASN 375: (O) O	O H

2-[(3-Methylbutoxy)carbonyl] benzoic acid	−4.4	3	2.106 1.896 1.979	ARG 374: (H) HH21 ARG 374: (H) HH12 ASN 375: (H) H	O O O

2-(5-Methylhexyl) benzoic acid	−3.43	1	2.044	ASN 375: (H) H	O

3-(Iminomethyl)-2,4-dimethylphenol	−3.23	2	2.226 1.700	ASN 375: (H) H ASN 375: (O) O	O O

Glide score, number of H-bond interactions, distance of H-bond interaction, and involved protein atom and ligand atom in interaction of isolated and standard drugs with COX1 protein obtained from docking studies.

**Table 6:** Docking score and H-bond interaction of ligands with COX2 protein (3LN1).

Ligand	*G* score	Number of H-bonds	Distance (Å)	Protein atom	Ligand atom
Diclofenac	−3.4	3	2.151 2.121 1.802	LYS543: (H) HZ3 LYS543: (H) HZ2 THR547: (H) HG1	(O) O3 O (O) O3

Indomethacin	−3.2	2	2.041 1.767	ARG297: (H) H LYS543: (H) HZ2	O (O) O4

Aspirin	−2.8	3	2.112 2.140 2.049	LYS 543: (H) HZ3 THR547: (H) HG1 ARG297: (H) H21	(O) O20 (O) O20 (O) O3

Paracetamol	−2.4	3	2.383 2.264 2.014	GLU539: (O) OE2 TRP531: (H) HE1 GLU539: (O) OE1	H O H

Morphine	−1.0	5	2.471 2.249 2.025 1.989 1.974	ARG297: (H) HE ASN556: (O) OD1 ARG297: (H) HH21 ARG297: (H) HH21 ARG297: (H) HH22	(O) O2 (H) H38 (O) O21 (O) O2 (O) O3

2-[(3-Methylbutoxy)carbonyl] benzoic acid	−2.9	3	2.115 2.065 1.774	HID228: (H) HD1 LYS 543: (H) HZ1 LYS 543: (H) HZ1	(O) O8 (O) OS2 (O) O13

1-(2-Hydroxyphenyl)-4-methylpentan-1-one	−2.4	2	2.085 1.726	TRP531: (H) HE1 ASP348: (O) OD1	(O) O7 H

3-Methylbutyl benzoate	−2.1	1	1.898	THR547: (H) HG1	O

4-Methylpentyl benzoate	−2.1	2	2.139 2.033	LYS543: (H) HZ2 THR547: (H) HG1	(O) O35 O

2-(5-Methylhexyl) benzoic acid	−1.3	1	2.156	TRP531: (H) HE1	O

3-(Iminomethyl)-2,4-dimethylphenol	−0.4	1	2.171	ASN546: (H) HD21	O

Glide score, number of H-bond interactions, distance of H-bond interaction, and involved protein atom and ligand atom in interaction of isolated and standard drugs with COX2 protein obtained from docking studies.
